# The use of fondaparinux and rituximab for recurrent thrombotic events in antiphospholipid syndrome

**DOI:** 10.1177/09612033221122147

**Published:** 2022-09-08

**Authors:** Zara Sayar, Shoshana Burke, Prabal Mittal, Hannah Cohen

**Affiliations:** 1 Department of Haematology, 9687 University College London Hospitals NHS Foundation Trust, London, UK; 2 Department of Haematology, Whittington Health NHS Trust, London, UK; 3 Haemostasis Research Unit, Department of Haematology, 4919 University College London, London, UK

**Keywords:** fondaparinux, recurrent thrombosis, venous thromboembolism, arterial thrombosis, antiphospholipid syndrome

## Abstract

Limited evidence exists to guide the management of recurrent thrombosis occurring despite therapeutic anticoagulation in patients with thrombotic antiphospholipid syndrome (APS). In this case series, fondaparinux, with or without an antiplatelet agent, provided an effective and safe option in three patients with thrombotic APS, all two triple and one single positive for antiphospholipid antibodies, who had recurrent venous and/or arterial thromboembolism. Rituximab was also used in all patients. Recurrent events occurred despite therapeutic anticoagulation, including at high-intensity, with warfarin and subsequent low-molecular-weight heparin. There were no major bleeding events. Adjunctive therapies used for thrombosis included catheter-directed thrombolysis and rituximab.

## Introduction

Rates of recurrent thrombosis in specific cohorts, rather than in the general population, vary. Antiphospholipid syndrome (APS) is characterised by thrombotic (venous, arterial and/or microvascular) and/or obstetric morbidity in association with persistently positive antiphospholipid antibodies (aPL; lupus anticoagulant [LA], IgG and/or IgM anti–beta-2 glycoprotein-1 [aβ2GP1] and anticardiolipin antibodies [aCL]).^1^ Triple aPL-positive APS patients (i.e. who have LA, IgG and/or IgM anti–beta-2 glycoprotein-1 and anticardiolipin antibodies) are at particularly high risk for recurrent thrombosis.^2^ Annualised recurrent thrombosis rates in patients with APS on vitamin K antagonist (VKA) were 1·3–4·0% in two prospective randomised controlled trials^3,4^ and 4·3% and 4·8% in a prospective^5^ and retrospective cohort study, respectively, the latter in triple aPL-positive patients.^2^ In a systematic review of 728 patients with APS treated with a direct oral anticoagulant (DOAC), 119 of 246 (48·3%) were triple aPL-positive, with the approximate annualised recurrent thrombosis rate 11%.^6^ Recurrent venous and/or arterial thromboembolism while on therapeutic anticoagulation is observed in several conditions other than APS, including cancer.

If a patient develops anticoagulant-refractory thrombosis, it is important to objectively confirm this by appropriate imaging techniques. Adherence to anticoagulation should be assessed. Alternative causes of thrombosis should be considered, such as the development of heparin-induced thrombocytopenia^7^ or malignancy^8^ such as myeloproliferative neoplasms or monoclonal gammopathy of undetermined significance (MGUS). Additional risk factors should be addressed, including the use of oestrogen-containing preparations.^9^ A review of bleeding risk factors should also be considered, with active management of any identified factors, to facilitate optimal antithrombotic treatment. Recurrent thromboses that occur due to a subtherapeutic International Normalised Ratio (INR) could be related to non-adherence or a spurious therapeutic INR due to LA effect on thromboplastin.^10^

The management of anticoagulant-refractory thrombosis can be challenging, and current guidelines are largely extrapolated from cancer cohorts.^11,12^ If further thrombotic events occur, despite the use of either high-intensity warfarin (INR target 3.5, range 3.0–4.0) or low-molecular-weight heparin (LMWH), evidence regarding further escalation of anticoagulation is limited. Fondaparinux is a synthetic analogue of heparin pentasaccharide required for antithrombin binding. It is mainly used in the treatment of heparin-induced thrombocytopenia^7^ and has specific anti-factor Xa activity that is seven times higher than LMWH. It is eliminated exclusively by the kidneys with an elimination half-life of 17 h,^13^ and therefore contraindicated in severe renal impairment.^13^ Two patients with APS treated with fondaparinux and mycophenolate mofetil have been reported to have favourable outcomes with no recurrent events in a 4-year follow-up period.^14^ This may be an option in recurrent thrombotic events with or without the addition of an antiplatelet agent.^15^

Here, we review fondaparinux and rituximab use in a single centre, for recurrent thrombotic events despite therapeutic warfarin and/or LMWH.

## Methods

Patients receiving fondaparinux for recurrent thrombosis were identified from the complex anticoagulation clinic at University College Hospital (UCH), London. A retrospective review of the notes was performed to identify patients with thrombotic APS who had experienced break-through thrombosis while on anticoagulation. Data were collected on thrombotic events and their management, including the use of adjunctive therapies to manage thrombosis. Written informed consent was obtained from the patients included in this case series.

## Results and discussion

Thrombotic histories and anticoagulation treatment are detailed in Table 1.

**Table 1.** Thrombotic histories, anticoagulation treatment and additional therapeutic modalities

Patient details:	Approximate CrCl (C&G) (ml/min)	Weight range (kg)	Underlying thrombotic diagnosis	Initial thrombosis (0 months)	1^st^ re-thrombosis and treatment	2^nd^ re-thrombosis and treatment	3^rd^ re-thrombosis and treatment	4^th^ re-thrombosis and treatment	5^th^ re-thrombosis and treatment
Patient 1: 28-year-old female	112	82–97	Thrombotic APS; Triple aPL-positive: IgG aβ2GPI 37.3 U/mL IgM aβ2GPI 28.4 U/mL IgG aCL 22.1U/mL IgM aCL neg^a^ TVT dilute phospholipid ratio 1.37 (NR: 0.8–1.2) Right popliteal DVT aged 28 years, week 20 of 1^st^ pregnancy (LMWH until 6w postpartum) Obstetric APS aged 30 years: Pre-eclampsia 28 weeks’ gestation	Left popliteal DVT, aged 40y Warfarin target 2.5, (range 2.0–3.0) APS diagnosed	4 months Extension of DVT to left common femoral vein while INR 2.5 Warfarin target INR increased to 3.5 (range 3.0–4.0)	12 months DVT extension within common femoral vein, also involving external iliac vein while INR 3.4 ∼20% increase on standard treatment dose LMWH	24 months Left sigmoid sinus thrombosis Acute frontal infarct following MRA ∼30% increase on standard treatment dose LMWH	36 months Segmental PE Course of rituximab Switched to fondaparinux 7.5 mg od	
Patient 2: 42-year-old male	127	116–124	Thrombotic APS: Arterial and venous thrombosis; triple aPL-positive: IgG aβ2GPI >100U/mL IgM aβ2GPI neg IgG aCL 49.4U/mL IgM aCL neg^a^ TVT ratio 1.54 (NR 0.80–1.12) ECT ratio 1.01 (NR 0.87–1.14) TVT/ECT % correction 34.4 INR 3.58	Left MCA infarct: Initial standard treatment with antiplatelet agents	1 month Right MCA infarct Triple aPL-positive identified Warfarin target INR 3.5 (range 3.0–4.0) initiated	21 months Right popliteal vein DVT (INR 2.6 at time of event) Continued warfarin (target INR 3.5)	23 months Proximal extension of DVT to superficial femoral vein (INR 2.2 at time of event) Standard treatment dose LMWH	23.5 months Segmental PEs ∼20% increase on standard treatment dose LMWH	26 months Proximal DVT extension to common femoral vein Switched to fondaparinux 5 mg bd Course of rituximab
Patient 3: 31-year-old female	101	76–94	Thrombotic APS: Arterial and venous thrombosis; IgG aβ2GPI 96.8U/mL^a^ IgM aβ2GPI neg IgG aCL neg IgM aCL neg LA negative	Cerebral venous sinus thrombosis Warfarin target INR 2.5 (range 2.0–3.0)	5 months Cerebral venous sinus thrombosis (very erratic INRs) Standard treatment dose LMWH	14 months Multiple anterior and posterior arterial circulation infarcts ∼20% increase on standard treatment dose LMWH IVC filter inserted Course of rituximab	24 months Bilateral iliofemoral + IVC thrombus with renal vein thrombosis Catheter-directed thrombolysis Fondaparinux 5 mg bd and clopidogrel started		

Abbreviations: aPL: antiphospholipid antibodies; APS: antiphospholipid syndrome; bd: twice daily; CrCl (C&G): Cockcroft and Gault creatinine clearance; DVT: deep venous thrombosis; ECT: ecarin time; IJV: internal jugular vein; INR: international normalised ratio (all venous); IVC: inferior vena cava; LMWH: low-molecular–weight heparin; m: month; MCA: middle cerebral artery; MRA: magnetic resonance angiography; neg: negative; NR: normal range; od: once daily; PE: pulmonary embolism; PICC: peripherally inserted central catheter; triple positive: presence of lupus anticoagulant, TVT: taipan venom time; IgG/IgM: anticardiolipin and anti-β2 glycoprotein I antibodies; w: weeks; y: years.

^a^Medium or high titre is defined as >40 GPL or MPL or >99th percentile, and/or aβ2GPI (IgG and/or IgM) >99th percentile^1^.

Three patients (two females, one male), median age 31 (range 28–42) years were on long-term fondaparinux. Two patients had triple aPL-positive thrombotic APS and one was single aPL-positive. Patient 1 also had a history of obstetric APS. No patients had a diagnosis of malignancy. Patients 1 and 3 were tested for monoclonal gammopathy^16^ and JAK2 V617F,^17^ neither of which were present. Patient 2 has not been tested for these.

The initial thrombotic event was venous in two patients: deep vein thrombosis (DVT) and cerebral venous sinus thrombosis. Treatment of these events was standard-intensity warfarin (INR target 2.5, range 2.0–3.0). Further thrombotic events in these patients included arterial (Patients 1 and 3) and venous only in Patient 2. One patient presented with an arterial event (middle cerebral artery [MCA] infarct), managed initially with antiplatelet treatment alone. A further contralateral MCA infarct and subsequent diagnosis of APS led to a change in antithrombotic treatment to high-intensity warfarin (INR target 3.5, range 3.0–4.0).^18,19^ Subsequent recurrent thrombotic events in this patient were venous.

**Figure 1.**
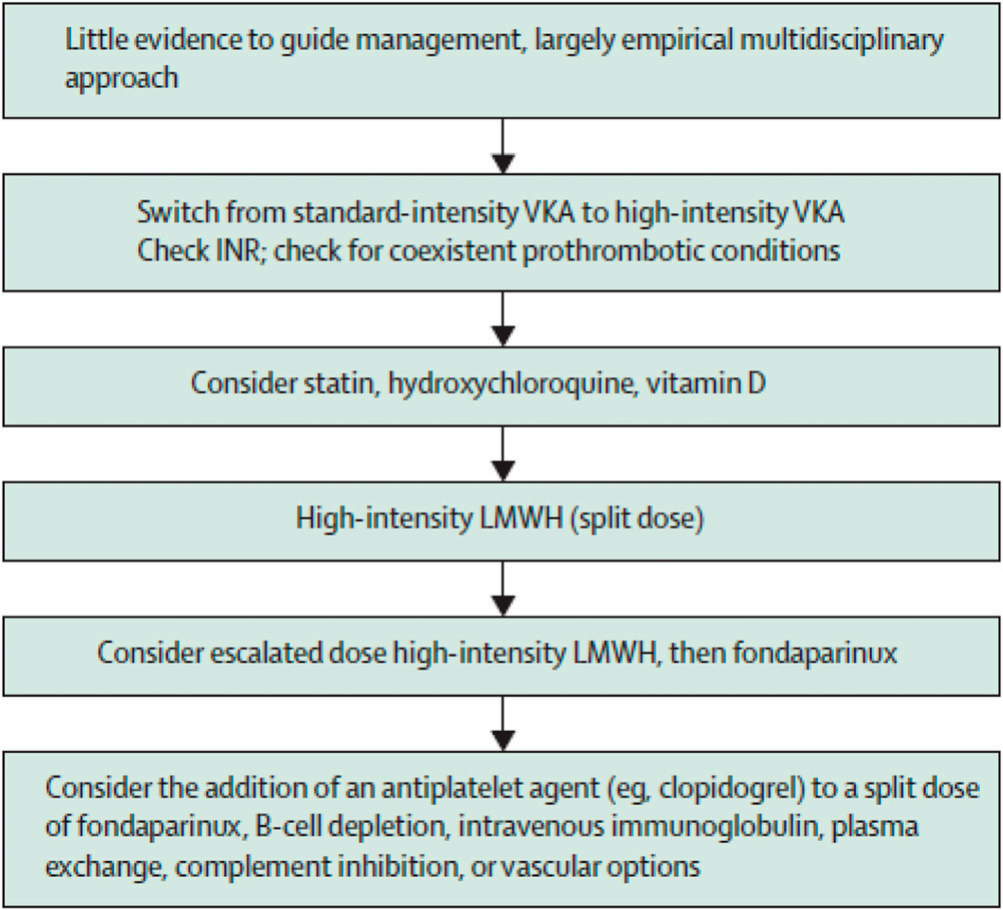
Proposed management for anticoagulant-refractory thrombotic antiphospholipid syndrome (Ref:^22^ Reproduced from Cohen et al). Abbreviations: LMWH, low-molecular–weight heparin; VKA, vitamin K antagonist.

Initial management of recurrent thrombosis included assessment of anticoagulation intensity at the time of the event and patient adherence. In all three cases, LMWH was used up to approximately 30% above standard treatment dose for further thrombotic events, prior to switching to standard therapeutic dose fondaparinux (see Figure 1). Patient 1 received split-dose high-intensity dalteparin, approximately 20% above standard treatment dose, after recurrent DVT while on warfarin as the INR was 3.4 at the time of re-thrombosis. Patient 3 received clopidogrel together with fondaparinux, split dose, as additional antithrombotic treatment. There were no subsequent thrombotic events on fondaparinux plus clopidogrel in patient 3. There were no major or clinically relevant non-major bleeding events. The median follow-up on fondaparinux was 70 (range 45–81) months.

Two patients in this case series have triple aPL-positive APS supporting that these patients are at particularly high thrombotic risk. In a retrospective study of the clinical course of 123 triple aPL-patients receiving long-term anticoagulation (mean follow-up 6.0 (±4.6) years, 36/123 (16 venous, 20 arterial) developed subsequent thromboembolism.^2^ The median INR at the time of these events was 2.3.

Two patients experienced a recurrent arterial event after an initial venous thrombotic event (Patient 1 and 3); and the third (patient 2), a venous event after an initial arterial event. In a study on the sites of recurrent thrombosis that follows an initial event in APS patients on VKA (N = 70), arterial events were followed by arterial events, and venous events by venous in 91% (49/54).^20^ A further study of 147 APS patients reported rates of 93% (89/96) and 76% (69/90) of patients, respectively.^21^ Thus, in most cases of patients treated with VKA, arterial and venous events “bred true,” unlike in this case series.

All three APS patients received a course of rituximab (375 mg/m^2^ × 4 weekly doses) as empirical treatment for anticoagulant-refractory thrombosis. The 16^th^ International Congress on Antiphospholipid Antibodies Treatment Trends Task Force suggests that rituximab may have a role in the treatment of some aPL-related non-criteria manifestations and refractory catastrophic APS; and notes the paucity of evidence to inform the use of rituximab for anticoagulant-refractory thrombotic APS,^18^ with case reports documenting its use.^22^

Catheter-directed thrombolysis (CDT) was used in one patient in the acute situation in our case series with an iliofemoral DVT. In the catheter-directed Venous Thrombolysis in acute iliofemoral vein thrombosis (CaVenT) randomised controlled trial that compared the short-term efficacy of CDT with standard treatment (anticoagulation and elastic compression stockings) in 103 patients, iliofemoral patency after six months was 64% in the CDT arm and 36% in the standard treatment arm.^23^

Our case series reports on the use of fondaparinux and rituximab for recurrent thrombosis in patients with APS; the generalisability of our results to other conditions associated with recurrent thrombosis (e.g. cancer) is undefined. Anticoagulation for recurrent thrombosis can be summarised thus (Figure 1): if recurrent thrombosis occurs while on a DOAC, a switch to LMWH or to VKA should be considered.^22^ For thrombosis that occurs while on standard therapeutic dose LMWH, the dose may be escalated by 25% and 33% sequentially.^15,22^ For recurrent thrombosis while taking standard-intensity VKA (INR target range 2.0–3.0), the target INR may be increased to high-intensity VKA (INR target range 3.0–4.0).

## Conclusion

In this small case series, fondaparinux provided effective and safe anticoagulation in three APS patients, who experienced recurrent arterial and/or venous thrombosis despite therapeutic anticoagulation, including at high-intensity, with warfarin and subsequent LMWH. Rituximab was used in all patients.
